# The association of exercise test variables with long-term mortality in patients with chronic Chagas disease

**DOI:** 10.3389/fmed.2022.972514

**Published:** 2022-09-20

**Authors:** Rudson S. Silva, Fernanda S. N. S. Mendes, Jerome L. Fleg, Luiz F. Rodrigues Junior, Marcelo C. Vieira, Isis G. G. Xavier, Henrique S. Costa, Michel S. Reis, Flavia Mazzoli-Rocha, Andrea R. Costa, Marcelo T. Holanda, Henrique H. Veloso, Gilberto M. Sperandio da Silva, Andréa S. Sousa, Roberto M. Saraiva, Alejandro Marcel Hasslocher-Moreno, Mauro F. F. Mediano

**Affiliations:** ^1^Evandro Chagas National Institute of Infectious Diseases, Oswaldo Cruz Foundation, Rio de Janeiro, RJ, Brazil; ^2^National Heart, Lung, and Blood Institute, National Institutes of Health, Bethesda, MD, United States; ^3^Department of Research and Education, National Institute of Cardiology, Rio de Janeiro, RJ, Brazil; ^4^Center for Cardiology and Exercise, Aloysio de Castro State Institute of Cardiology, Rio de Janeiro, RJ, Brazil; ^5^Physical Therapy Department, Federal University of Jequitinhonha and Mucuri Valleys, Diamantina, MG, Brazil; ^6^Faculty of Physical Therapy, School of Medicine, Federal University of Rio de Janeiro, Rio de Janeiro, RJ, Brazil

**Keywords:** Chagas disease, mortality, prognosis, VO_2_max, maximal functional capacity

## Abstract

**Background:**

The identification of variables obtained in the exercise test (ET) associated with increased risk of death is clinically relevant and would provide additional information for the management of Chagas disease (CD). The objective of the present study was to evaluate the association of ET variables with mortality in patients with chronic CD.

**Methods:**

This retrospective longitudinal observational study included 232 patients (median age 46.0 years; 50% women) with CD that were followed at the Evandro Chagas National Institute of Infectious Diseases (Rio de Janeiro, Brazil) and performed an ET between 1989 and 2000. The outcome of interest was all-cause mortality.

**Results:**

There were 103 deaths (44.4%) during a median follow-up of 21.5 years (IQR 25–75% 8.0–27.8), resulting in 24.5 per 1,000 patients/year incidence rate. The ET variables associated with mortality after adjustments for potential confounders were increased maximal (HR 1.02; 95% CI 1.00–1.03 per mmHg) and change (HR 1.03; 95% CI 1.01–1.06 per mmHg) of diastolic blood pressure (DBP) during ET, ventricular tachycardia at rest (HR 3.95; 95% CI 1.14–13.74), during exercise (HR 2.73; 95% CI 1.44–5.20), and recovery (HR 2.60; 95% CI 1.14–5.91), and premature ventricular complexes during recovery (HR 2.06; 1.33–3.21).

**Conclusion:**

Our findings suggest that ET provides important prognostic value for mortality risk assessment in patients with CD, with hemodynamic (increased DBP during exercise) and electrocardiographic (presence of ventricular arrhythmias) variables independently associated with an increased mortality risk in patients with CD. The identification of individuals at higher mortality risk can facilitate the development of intervention strategies (e.g., close follow-up) that may potentially have an impact on the longevity of patients with CD.

## Introduction

Chagas disease (CD) is an important public health problem that affects ~6 million people worldwide, most of them living in Latin America, but also with cases in United States, Europe, and Oceania (1, 2). It is responsible for loss of more than 800,000 disability-adjusted life-years and a global economic loss of ~US$ 850 million per year (3). The cardiac form, also known as chronic Chagas cardiomyopathy (CCC), is considered the most severe clinical presentation of chronic CD, being responsible for the highest morbidity and mortality rates associated with CD. CCC is characterized by decreased functional capacity, arrhythmias, thromboembolic events, and heart failure (4–6).

The exercise test (ET) is an important tool to assess functional capacity by estimated maximal oxygen uptake (VO_2_max), heart rhythm and hemodynamic variables during exercise, being associated with prognosis in both healthy individuals and patients with a variety of chronic conditions, with greater functional capacity associated with lower mortality rates (7–9). It is a feasible and low-cost evaluation method that can be easily implemented in clinical practice. Although ET is a well-recognized tool for risk stratification of patients with a wide variety of clinical conditions, studies evaluating the prognostic value of ET in patients with CD remain scarce, with most of them present conflicting results and limited by short-term follow-up, small sample sizes, and inclusion of exclusively patients with CCC (10).

The identification of mortality predictors obtained in the ET is clinically relevant and may provide additional information for the management of patients with CD (11, 12). Therefore, the objective of this study was to evaluate the association of ET variables with long-term mortality rates in patients with chronic CD. We hypothesized that ET could provide important prognostic information among individuals with CD, with decreased exercise capacity measured by estimated VO_2_max and presence of arrhythmias during exercise being associated with an increased risk of death.

## Methods

This is a retrospective longitudinal observational study including patients with CD who were followed at the Evandro Chagas National Institute of Infectious Disease (INI/Fiocruz) outpatient clinic and underwent an ET between 1989 and 2000. The diagnosis of CD was made in the immunodiagnostic sector of INI/Fiocruz using the following tests: indirect immunofluorescence- IFI (Bio-Manguinhos, Fiocruz) and enzyme linked immunosorbent assay—ELISA (Wiener lab—Chagatest), both commercial tests. CD diagnosis was confirmed when both IFI and ELISA were reactive. The INI/Fiocruz is a national referral center for screening, treatment and research of infectious and tropical diseases in Brazil. Patients were admitted by spontaneous demand or were referred from other healthcare institutions or blood banks. At the time of enrollment of patient in the INI/Fiocruz clinic, participant underwent an initial evaluation protocol, which included clinical and epidemiological anamnesis, a physical examination focused on chronic CD-related cardiovascular and digestive signs and symptoms, a 12-lead electrocardiogram and a two-dimensional Doppler echocardiogram. Participants were indicated to perform ET according to the physician discretion during their medical appointments. Those who did not undergo at least one medical appointment after the ET were excluded from the analysis. This study was approved by the INI/Fiocruz Research Ethics Committee (CAAE: 27576620.0.0000.5262). Patients signed consent was waived due to the retrospective nature of the study.

### Exercise test

ET was preferentially performed using a treadmill, but patients with any walking limitation (e.g., osteoarticular pain) were advised to perform the test on the cycle ergometer. The ET protocols used during the treadmill tests were Bruce, Naughton, and Astrand, whereas the ET protocols used during the cycle ergometer tests were Bruce, Ramp, and Jones. The choice of ET protocol took into consideration the clinical and functional condition of each patient in order to achieve the maximal exercise effort in a period between 8 and 12 min (8).

Variables of interest were collected using the ET physical forms of each patient. The electrocardiographic findings were continuously assessed at rest for 5 min, before starting the ET, throughout ET and during 5 min of recovery to detect ST-segment abnormalities, premature atrial contractions (PACs), supraventricular tachycardia (SVT), premature ventricular complexes (PVCs), and ventricular tachycardia (VT) considering together sustained and non-sustained tachycardia, classified according to the Minnesota code (13, 14). Heart rate (HR) was recorded at rest, during maximal exercise (HRmax) and after 1-min recovery (HRrec). Change of HR during ET (ΔHR during exercise) was obtained subtracting HRmax by resting HR. The chronotropic deficit was determined by the inability to reach 85% of HRmax predicted for age following the Karvonen formula (220-age) (15). A reduction in the peak exercise HR ≤ 12 beats after 1-min recovery was used to define decreased parasympathetic activity (16). Measures of systolic (SBP) and diastolic (DBP) blood pressure were obtained at rest, during peak exercise and through 5 min of recovery after exercise cessation. Change of SBP (ΔSBP during Exercise) and DBP (ΔDBP during Exercise) during ET was obtained subtracting maximal SBP and DBP by their respective resting values. The double product was calculated as the product of the SBP times HR at the peak of ET. The VO_2_max (mL.kg^−1^.min^−1^) was indirectly estimated by a specific formula depending on the ergometer and the protocol used during the test (17–21). The level of metabolic equivalents achieved (MET) at the peak of exertion was calculated as a multiple of the basal metabolic rate. Functional aerobic impairment (FAI) was calculated as the difference between the estimated and expected functional capacity for the age and sex, expressed in percentage. Categories of cardiorespiratory fitness (CF) according to age and sex were determined following the American Heart Association classification (22).

### Outcome

The outcome of interest was all-cause mortality. Death status was ascertained until December 31st, 2021 using personal information (patient's and parents' name, date of birth, or the number of federal identification document) through review of the INI/Fiocruz electronic medical records, online consultation of the mortality database system of the Department of Justice and Department of Health (SES/RJ) from the State of Rio de Janeiro, and through telephone contact with patients or their relatives when available. Follow-up period was measured from the date of the ET until the occurrence of event (death) or the last medical appointment, including those lost to follow-up, at which point follow-up was administratively censored.

### Covariates

Covariates were collected from INI/Fiocruz electronic medical records including demographic and clinical information at the time of ET. The main covariates obtained from medical records comprised age, sex, self-reported race, region of origin according to prevalence of CD, CD classification, left ventricular ejection fraction (LVEF), and comorbidities including arterial hypertension, diabetes mellitus, dyslipidemia, heart failure, stroke, and non-CD cardiomyopathy. Information about the region of origin considered the prevalence of chronic CD in Brazil, which was based on serological data from national surveys, being categorized as low (<2%), medium (2–4%), high (>4%), and non-endemic (States of Rio de Janeiro and Espírito Santo). The classification of CD (indeterminate, cardiac, and digestive forms) and the stages of cardiac form followed the recommendations from the 2^nd^ Brazilian Consensus on Chagas disease (5). To facilitate data analysis and following a clinical rationale, patients were grouped as following: indeterminate (positive CD serology test without any CD-specific electrocardiographic and echocardiographic abnormalities), CCC without heart failure (positive CD serology test and CD-specific electrocardiographic and/or echocardiographic abnormalities but without symptoms of heart failure), CCC with heart failure (positive CD serology test and CD-specific electrocardiographic and/or echocardiographic abnormalities with symptoms of heart failure), and/or digestive forms (presence of esophagus and/or intestine peristalsis dysfunction). LVEF was measured with transthoracic echocardiography using the Teicholz method by a single trained evaluator within 6 months before or after the ET.

### Statistical analysis

Continuous data were presented as median (interquartile range 25–75%) and categorical variables were presented as number of observations (percentages). Baseline comparisons between groups of patients were performed using the Mann-Whitney test for continuous and the Chi-squared test for categorical variables. Cumulative incidence (expressed by the number of patients that presented the outcome divided by the total number of patients during the follow-up) and the incidence rate (expressed as the number of patients that presented the outcome divided by the time of exposure of each individual) were calculated in the incidence analysis. Cox proportional hazards models were used to estimate hazard ratios (HRs) and 95% confidence intervals (CI) for the association between ET variables and death. Models were fitted without adjustments, as well as adjusted for potential confounders including age, sex, race, CD classification (indeterminate/digestive, CCC without heart failure, and CCC with heart failure), LVEF, and presence of comorbidities (hypertension, diabetes mellitus, dyslipidemia, non-CD cardiomyopathy, or stroke). Schoenfeld residuals tests were performed to evaluate proportional hazard assumptions. The predictive value of Cox survival models was assessed using the Harrell's C indexes. The variance inflation factor (VIF) for the covariates included in the adjusted models was 1.22, suggesting no multicollinearity problems. Sensitivity analyses including the type of ET ergometer (treadmill or cycle ergometer) as a covariate were also conducted.

Kaplan-Meier survival curves were constructed and compared using the log-rank test. Data analyses were performed using Stata 13.0 statistical software (College Station, TX: StataCorp LP) with 2-tailed significance level of *p* ≤ 0.05 for all analyses.

## Results

Of the 235 eligible patients, 3 were excluded following the pre-defined exclusion criteria, leaving a final sample of 232 patients. The characteristics of the study sample are depicted in Tables 1, 2. The median age was 46.0 years. There was a balanced distribution of sex (50% women). White (54.3%) was the most common self-reported race. Most patients (48.7%) presented the indeterminate form of CD, followed by those with CCC without heart failure (45.7%). The median LVEF was 64.0%. The median time between INI-Fiocruz enrollment and ET performance was 0.6 years (IQR 25–75% 0.2–2.8). The median of estimated VO_2_max was 31.3 mL.kg^−1^.min^−1^, with most patients (52.1%) presenting a good or excellent CF classification based on age and sex. Most ET were performed on a treadmill (85.8%, *n* = 199), 95.0% (*n* = 189) of those under Bruce protocol. Median of estimated VO_2_max obtained from treadmill ET was slightly higher in comparison to those obtained on the cycle ergometer (31.5 vs. 30.0 mL.kg^−1^.min^−1^, respectively; *p* = 0.05), with a similar distribution of age (*p* = 0.62) and sex (*p* = 0.19). ST-segment abnormalities were observed in 8 patients (3.5%), of those 6 ST-segment depressions, 1 early repolarization, and 1 T-wave inversion. One-third of patients (34.1%) had arrhythmias at rest [6.5% PACs, 29.7% PVCs, and 2.2% VT (1.7% non-sustained VT and 0.5% sustained VT)], whereas 56.9% presented arrhythmias during effort [15.1% PACs, 0.4% SVT, 50.4% PVCs, and 9.5% VT (8.2% non-sustained VT and 1.7% sustained VT)], and 44.4% had arrhythmias during the recovery period [12.5% PACs, 0.9% SVT, 36.6% PVCs, and 6.5% VT (5.2% non-sustained VT and 1.3% sustained VT)].

**Table 1:** Baseline demographic and clinical characteristics of patients (total and stratified by survival status) (*n* = 232).

**Variable**	**Median (IQR 25–75%) or frequency (%)**			
	**Total**	**Deceased**	**Alive**	*p*-value^*^
		**(*n* = 103; 44.4%)**	**(*n* = 129; 55.6%)**	
Age (years)	46.0 (39.0–52.0)	49.0 (43.0– 55.0)	43.0 (35.0–50.0)	**<0.001**
Women	116 (50.0)	50 (48.5)	66 (51.2)	0.692
**Race**				
White	126 (54.3)	64 (62.1)	62 (48.1)	**0.033**
Non-white	106 (45.7)	39 (37.9)	67 (51.9)	
**Region of origin according to prevalence**				
Non-endemic Chagas disease area	11 (4.8)	5 (3.9)	6 (5.9)	0.477
Low Chagas disease prevalence area	12 (5.2)	9 (7.0)	3 (2.9)	0.170
Medium Chagas disease prevalence area	72 (31.2)	35 (27.1)	37 (36.3)	0.136
High Chagas disease prevalence area	136 (58.9)	80 (62.0)	56 (54.9)	0.275
Body mass index (Kg/m^2^) (*n* = 148)	24.7 (22.4–27.5)	24.1 (23.0–26.9) *n* = 57	25.6 (22.3–27.7) *n* = 91	0.329
Arterial hypertension	57 (24.6)	29 (28.2)	28 (21.7)	0.257
Diabetes mellitus	4 (1.7)	4 (3.9)	0 (0.0)	**0.024**
Dyslipidemia	37 (16.0)	15 (14.6)	22 (17.0)	0.607
Non-CD cardiomyopathy	9 (3.9)	4 (3.9)	5 (3.9)	0.998
Heart failure	13 (5.6)	10 (9.7)	3 (2.3)	**0.015**
Stroke	1 (0.4)	1 (1.0)	0 (0.0)	0.262
**Clinical presentation of CD**				
Indeterminate	113 (48.7)	42 (40.8)	71 (55.0)	**0.031**
Cardiac without CCC	106 (45.7)	51 (49.5)	55 (42.6)	0.296
Cardiac with CCC	13 (5.6)	10 (9.7)	3 (2.3)	**0.015**
Digestive	5 (2.2)	3 (2.9)	2 (1.5)	0.478

CCC, chronic Chagas cardiomyopathy; VO_2_, Oxygen Consumption; AHA, American Heart Association; ΔHR, heart rate change; ΔSBP, Systolic Blood Pressure Change; ΔDBP, Diastolic Blood Pressure Change; LVEF, Left Ventricular Ejection Fraction.

Estimates **in bold** were statistically significant.

**Table 2:** Exercise test characteristics of patients (total and stratified by survival status) (*n* = 232).

**Variable**	**Median (IQR 25–75%) or frequency (%)**			
	**Total**	**Deceased**	**Alive**	*p*-value^*^
		**(*n* = 103; 44.4%)**	**(*n* = 129; 55.6%)**	
VO_2_max (mL.kg^−1^.min^−1^)	31.3 (24.6–39.3)	30.8 (24.6–37.5)	34.1 (27.9–41.4)	**0.002**
FAI (%)	0.9 (−15.7 to 16.9)	4.7 (−14.7 to 22.5)	−1.5 (−15.7 to 14.8)	0.446
METs	8.9 (7.0–11.3)	8.7 (7.0–10.7)	9.7 (8.0–11.8)	**0.001**
Resting HR (bpm)	75.0 (67.0–85.0)	73.0 (65.0–82.0)	77.0 (69.0–87.0)	**0.027**
Maximal HR (bpm)	155.0 (138.0–170.0)	150.0 (130.0–165.0)	160.0 (141.0–173.0)	**0.008**
ΔHR during exercise	78.0 (59.0–93.0)	77.0 (57.0–90.0)	79.0 (60.0–94.0)	0.224
Chronotropic deficit (HRmax <85%)	84 (36.2)	38 (36.9)	46 (35.7)	0.846
Recovery HR at 1^st^minute (bpm)	121.0 (104.0–137.0)	121.0 (103.0–135.0)	121.0 (105.0–141.0)	0.358
ΔHR during recovery at 1^st^ minute (bpm)	−30.0 (−41.0 to −21.0)	−29.0 (−38.0 to −20.4)	−32.0 (−44.0 to −22.0)	0.099
ΔHR during recovery ≤ 12 bpm	19 (8.3)	11 (10.9)	8 (6.3)	0.213
Resting SBP (mmHg)	120.0 (110.0–140.0)	130.0 (120.0–140.0)	120.0 (110.0–130.0)	0.064
Resting DBP (mmHg)	80.0 (80.0–90.0)	80.0 (80.0–90.0)	80.0 (70.0–90.0)	0.327
Maximal SBP (mmHg)	167.5 (150.0–182.5)	170.0 (150.0–195.0)	160.0 (150.0–180.0)	0.393
Maximal DBP (mmHg)	80.0 (80.0–90.0)	82.5 (80.0–90.0)	80.0 (75.0–90.0)	**0.006**
ΔSBP during exercise (mmHg)	40.0 (30.0–60.0)	40.0 (30.0–60.0)	40.0 (30.0–60.0)	0.960
ΔDBP during exercise (mmHg)	0.0 (0.0–10.0)	0.0 (0.0–10.0)	0.0 (−5.0 to 10.0)	0.088
Double Product (mmHg.bpm)	26,000 (21,000–30,000)	25,500 (20,720–29,520)	26,250 (21,920–30,240)	0.371
**Cardiorespiratory fitness level (AHA)**				
Very poor and poor	43 (18.5)	23 (22.3)	20 (15.5)	0.184
Regular	68 (29.3)	34 (33.0)	34 (26.4)	0.269
Good and excellent	121 (52.1)	46 (44.7)	75 (58.1)	**0.041**
LVEF (Teicholz, %)	64.0 (58.0–69.0)	63.0 (50.0–68.0)	65.0 (61.0–69.0)	**0.010**
ST-segment abnormalities	8 (3.5)	3 (2.9)	5 (3.9)	0.689
**Supraventricular arrhythmia**				
Resting PACs	15 (6.5)	8 (7.8)	7 (5.4)	0.471
Resting SVT	0 (0.0)	0 (0.0)	0 (0.0)	1.00
Exercise PACs	35 (15.1)	15 (14.6)	20 (15.5)	0.842
Exercise SVT	1 (0.4)	1 (1.0)	0 (0.0)	0.262
Recovery PACs	29 (12.5)	16 (15.5)	13 (10.1)	0.212
Recovery SVT	2 (0.9)	1 (1.0)	1 (0.8)	0.873
**Ventricular arrhythmia**				
Resting PVCs	69 (29.7)	43 (41.7)	26 (20.2)	**<0.001**
Resting VT	5 (2.2)	4 (3.9)	1 (0.8)	0.105
Exercise PVCs	117 (50.4)	60 (58.2)	57 (44.2)	**0.033**
Exercise VT	22 (9.5)	15 (14.6)	7 (5.4)	**0.018**
Recovery PVCs	85 (36.6)	56 (54.4)	29 (22.5)	**<0.001**
Recovery VT	15 (6.5)	8 (7.8)	7 (5.4)	0.471

FAI, Functional Aerobic Impairment; SBP, Systolic Blood Pressure; DBP, Diastolic Blood Pressure; HR, Heart Rate; MET, Metabolic Equivalent; PACs, Premature atrial contractions; SVT, Supraventricular tachycardia; PVCs, Premature ventricular complexes; VT, Ventricular tachycardia.

Estimates **in bold** were statistically significant.

The comparison of baseline characteristics according to survival status is also depicted in Tables 1, 2. Patients who died were older (49.0 vs. 43.0 years; *p* < 0.001), more likely to be white (62.1 vs. 48.1%; *p* = 0.03), more likely to have diabetes (3.9 vs. 0.0%; *p* = 0.024) and heart failure (9.7 vs. 2.3%; *p* = 0.015), had a lower functional capacity (estimated VO_2_max 30.8 vs. 34.1 mL.kg^−1^.min^−1^; *p* = 0.01 and METs 8.7 vs. 9.7; *p* = 0.001), lower percentage of individuals with good and excellent cardiorespiratory fitness (44.7 vs. 55.8%; *p* = 0.04), and lower resting and maximal HR (73.0 vs. 77.0; *p* = 0.03 and 150.0 vs. 160.0 bpm; *p* = 0.008, respectively). LVEF was lower among those who died (63.0 vs. 65.0%; *p* = 0.01). Deceased individuals also had a greater percentage of arrhythmias at rest (47.6 vs. 23.3%; *p* < 0.001), during exercise (66.0 vs. 49.6%; *p* = 0.01) and recovery (63.1 vs. 29.5%; *p* < 0.001), with a higher frequency of resting PVCs (41.7 vs. 20.2%; *p* < 0.001), exercise PVCs (58.2 vs. 44.2%; *p* = 0.03) and VT (14.6 vs. 5.4%; *p* = 0.018), and recovery PVCs (54.4 vs. 22.5%; *p* < 0.001).

The associations between ET variables with death are depicted in Table 2. There were 103 deaths (44.4%) during a median follow-up of 21.5 years (IQR 25–75% 8.0–27.8), resulting in 24.5 per 1,000 patients/year incidence rate. The cause of death was due to heart failure (*n* = 17; 16.5%), sudden death (*n* = 26; 25.3%), stroke (*n* = 1; 1.0%), not related to CD (*n* = 19; 18.5%), and unknown (*n* = 40; 38.8%). Fifty-four patients (23.2%) were censored due to loss of follow-up. The characteristics of patients according to loss to follow-up status is demonstrated in Supplementary Table 1. Overall, losses to follow-up had a lower follow-up time, were younger, with a greater functional capacity, and lower percentage of resting PVCs. The median follow-up time of those lost to follow-up was 5.1 years.

**Figure 1:**
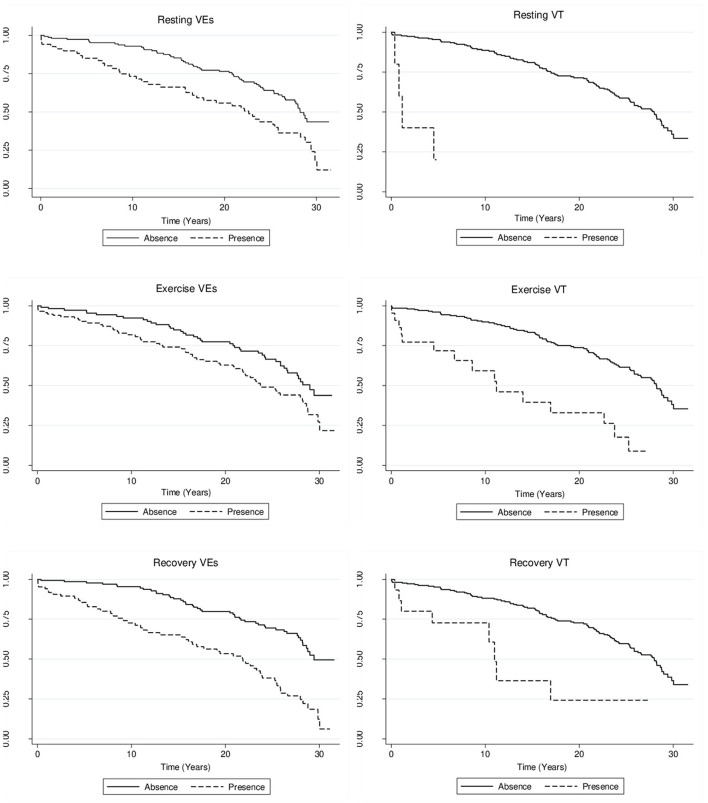
Unadjusted Kaplan-Meier curves for mortality by presence of ventricular arrhythmias.

The associations between ET variables and all-cause mortality are depicted in Table 3. Higher estimated VO_2_max was significantly associated with lower mortality rates in unadjusted analysis (HR 0.97; 95% CI 0.95–0.99 per mL.kg^−1^.min^−1^). However, this association was not further significant after adjustments for potential confounders. A greater mortality risk was observed among those with increased maximal DBP (HR 1.02; 95% CI 1.00–1.03 per mmHg; C-index 0.75) and ΔDBP (HR 1.03; 95% CI 1.01–1.06 per mmHg; C-index 0.75) during ET in the adjusted analysis. A higher mortality risk was observed among those with PACs during recovery (HR 1.78; 95% CI 1.04–3.04 per presence) in the unadjusted model, that did not remain statistically significant after adjustments for potential confounders. Ventricular arrhythmias (both PVCs and VT) at rest, during exercise and recovery were statistically associated with a greater mortality risk in the unadjusted analyses. However, only VT at rest (HR 3.95; 95% CI 1.14–13.74 per presence; C-index 0.74), during exercise (HR 2.73; 95% CI 1.44–5.20 per presence; C-index 0.75), and during recovery (HR 2.60; 95% CI 1.14–5.91 per presence; C-index 0.74), and PVCs during recovery (HR 2.06; 95% CI 1.33–3.21 per presence; C-index 0.75) maintained statistical significance after adjustments for potential confounders. The Harrel's C-index for the adjusted Cox models ranged from 0.73 to 0.75. Sensitivity analysis including the type of ET ergometer in the adjusted model provided similar estimates for all studied variables (Supplementary Table 2). The influence of resting, exercise, and recovery ventricular arrhythmias on mortality are illustrated in Kaplan-Meier Survival curves (Figure 1).

**Table 3:** Unadjusted and adjusted associations between ET variables and all-cause mortality.

	**Unadjusted**			Adjusted*			Harrel's C-index**
**Variable**	**HR**	**95% CI**	***p*-value**	**HR**	**95% CI**	***p*-value**	
VO_2_max (mL.kg^−1^.min^−1^)	**0.97**	**(0.95–0.99)**	**0.014**	0.98	(0.95–1.00)	0.158	0.73
FAI (%)	1.00	(0.99–1.01)	0.819	1.01	(1.00–1.02)	0.132	0.73
Resting HR (bpm)	0.99	(0.98–1.00)	0.175	1.00	(0.99–1.01)	0.930	0.73
Maximal HR (bpm)	0.99	(0.99–1.00)	0.123	1.00	(0.99–1.01)	0.778	0.73
ΔHR during exercise (bpm)	1.00	(0.99–1.00)	0.472	1.00	(0.99–1.01)	0.823	0.74
Chronotropic deficit (HRmax <85%)	0.98	(0.66–1.47)	0.936	0.94	(0.62–1.43)	0.773	0.74
Recovery HR at 1st minute (bpm)	1.00	(0.99–1.00)	0.512	1.00	(0.99–1.01)	0.443	0.74
ΔHR during recovery at 1st minute	1.01	(1.00–1.02)	0.133	1.00	(0.99–1.02)	0.432	0.74
ΔHR recovery ≤ 12 bpm	1.22	(0.65–2.28)	0.540	1.25	(0.65–2.40)	0.504	0.74
Resting SBP (mmHg)	1.01	(1.00–1.02)	0.161	1.00	(0.99–1.02)	0.364	0.74
Resting DBP (mmHg)	1.01	(0.99–1.03)	0.322	1.00	(0.98–1.02)	0.915	0.74
Maximal SBP (mmHg)	1.00	(0.99–1.01)	0.756	1.00	(0.99–1.01)	0.647	0.74
Maximal DBP (mmHg)	1.01	(1.00–1.02)	0.115	**1.02**	**(1.00**–**1.03)**	**0.040**	**0.75**
ΔSBP during exercise (mmHg)	0.99	(0.98–1.00)	0.230	0.99	(0.98–1.00)	0.372	0.74
ΔDBP during exercise (mmHg)	1.01	(0.99–1.03)	0.254	**1.03**	**(1.01–1.06)**	**0.006**	**0.75**
Double product (mmHg.bpm)	1.00	(0.99–1.00)	0.257	1.00	(0.99–1.00)	0.961	0.74
**Cardiorespiratory fitness level (AHA)**							
Very poor and poor	Reference			Reference			0.74
Regular	0.93	(0.55–1.58)	0.795	0.94	(0.54–1.62)	0.81	
Good and excellent	0.68	(0.41–1.12)	0.131	0.66	(0.39–1.12)	0.12	
ST-segment abnormalities	0.92	(0.29–2.91)	0.890	0.88	(0.27–2.82)	0.825	0.73
**Supraventricular arrhythmias**							
Resting PACs	1.91	(0.93–3.93)	0.079	1.42	(0.63–3.18)	0.394	0.74
Resting SVT^†^	–	–	–	–	–	–	–
Exercise PACs	1.00	(0.58–1.73)	1.000	0.98	(0.55–1.74)	0.951	0.74
Exercise SVT	2.12	(0.29–15.25)	0.456	1.69	(0.21–13.30)	0.617	0.74
Recovery PACs	**1.78**	**(1.04–3.04)**	**0.034**	1.21	(0.68–2.11)	0.495	0.74
Recovery SVT	1.64	(0.23–11.80)	0.624	1.35	(0.17–10.54)	0.775	0.74
**Ventricular arrhythmias**							
Resting PVCs	**1.98**	**(1.34–2.93)**	**0.001**	1.31	(0.84–2.02)	0.231	0.74
Resting VT	**26.89**	**(8.30–87.17)**	**<0.001**	**3.95**	**(1.14–13.74)**	**0.030**	0.74
Exercise PVCs	**1.66**	**(1.12–2.46)**	**0.011**	1.05	(0.69–1.60)	0.810	0.74
Exercise VT	**4.17**	**(2.37–7.33)**	**<0.001**	**2.73**	**(1.44–5.20)**	**0.002**	0.75
Recovery PVCs	**3.05**	**(2.06–4.51)**	**<0.001**	**2.06**	**(1.33–3.21)**	**0.001**	0.75
Recovery VT	**3.65**	**(1.74–7.63)**	**0.001**	**2.60**	**(1.14–5.91)**	**0.023**	0.74

VO_2_, Oxygen Consumption; FAI, Functional Aerobic Impairment; SBP, Systolic Blood Pressure; DBP, Diastolic Blood Pressure; HR, Heart Rate; MET, Metabolic Equivalent; AHA, American Heart Association; ΔHR, heart rate change; ΔSBP, Systolic Blood Pressure Change; ΔDBP, Diastolic Blood Pressure Change; LVEF, Left Ventricular Ejection Fraction; PACs, Premature atrial contractions; SVT, Supraventricular tachycardia; PVCs, Premature ventricular complexes; VT, Ventricular tachycardia.

*Cox regression model adjusted for age, sex, race, classification of CD (indeterminate, CCC without heart failure, and CCC with heart failure), LVEF, and presence of comorbidities (arterial hypertension, diabetes mellitus, dyslipidemia, non-CD cardiomyopathy, or stroke).

**Harrel's C-Index for the adjusted model.

^†^Non-valid HR estimates due to zero counts in at least one of the groups.

Estimates **in bold** were statistically significant.

## Discussion

The present study demonstrated an important prognostic value of ET in patients with CD, with DBP during exercise (both maximal and Δ), presence of VT (considering together sustained and non-sustained arrhythmias) at rest, during exercise, and recovery, and PVCs during recovery independently associated with high mortality rates in a long-term follow-up (median of 21 years). Surprisingly, higher estimated VO_2_max was not independently associated with a mortality risk reduction contrary to what we previously hypothesized.

ET is a widely used method to evaluate clinical, hemodynamic, and electrocardiographic responses to physical effort, providing important prognostic information in a variety of clinical conditions (7, 16, 23, 24). However, only few studies have been conducted in patients with CD, mostly limited by small sample sizes, short-term follow-up, and restricted to patients with CCC (10). A recent study including 49 CCC patients with impaired LVEF found that a decreased VO_2_max was significantly associated with an increased cardiovascular mortality during a mean follow-up period of 39 months. This study suggested a VO_2_ cut-point of 25 mL.kg^−1^.min^−1^, but with no significant difference for cardiovascular mortality between those below and above this cutoff point in the Kaplan Meier survival analysis (*p*-value = 0.36) (25). Conversely, a later prospective observational longitudinal study with 75 CCC patients conducted by the same research group did not find any significant association between VO_2_max and a composite outcome of cardiovascular events including cardiovascular death, heart transplantation, or ischemic event in a 41 months follow-up (26), similar to the findings observed in our study.

Studies that directly evaluated VO_2_max by gas exchange analysis during cardiopulmonary exercise testing (CPET) in patients with CCC demonstrated a beneficial association of higher VO_2_max and lower incidence of adverse health outcomes (27–29). However, CPET is a relatively expensive technique that requires specialized equipment and personnel, limiting its application in low socioeconomic settings, a common characteristic of patients with CD that live in endemic regions (30, 31). On the other hand, standard ET is a relatively low cost and accessible method that allows a reliable and inexpensive evaluation of hemodynamic and electrocardiographic responses to physical effort that can be easily implemented in clinical facilities located in low-income areas where CPET is not available.

The median estimated VO_2_max in the present study was higher in comparison to other studies that included CCC patients with similar age and sex distribution. This difference may be attributed to our inclusion of a large proportion (~50%) of patients with the indeterminate form of CD, without any structural cardiac disease that negatively impacts functional capacity, partially explaining the absence of association between VO_2_max and mortality. The results from the present study may provide a more reliable estimate about the prognostic effects of ET in the overall CD population, not limited to those patients with structural cardiomyopathy, considering that most patients in the overall CD population present the indeterminate form (32, 33).

A variable that was independently associated with greater mortality rate was an increased DBP during exercise. Sheps et al. (34) demonstrated that an abnormal DBP response during treadmill exercise was associated with a greater frequency of coronary artery disease and LV contraction abnormalities. However, other studies investigating the prognostic value of abnormal DBP responses to exercise did not demonstrate any significant association with mortality. For example, Sydó et al. (35) studied 20,000 patients with no previous history of cardiovascular diseases and found no association between DBP responses during the ET and mortality over an average follow-up of 12 years. This result is in accordance with those observed by Myers et al. (7) in healthy men and patients with cardiovascular diseases. Increased DBP responses to exercise may be a result of increased arterial stiffness and endothelial dysfunction, which are considered early signs of atherosclerotic vascular disease (36, 37). Considering the change in the epidemiological profile of patients with CD over recent decades, in which most of CD individuals migrated from rural to urban areas, facilitating their exposure to inadequate lifestyle habits not previously common in this population, it is reasonable to speculate that an exaggerated increase in DBP during exercise could be associated with early vascular abnormalities that increased their risk of death (38).

The results from the present study indicates that VT (considering together sustained and non-sustained arrhythmias) at rest, during exercise, and recovery were important independent mortality predictors in patients with CCC. Previous studies have demonstrated the adverse prognostic value of VT in patients with CCC, with non-sustained VT in a 24-h Holter monitoring being one of the variables included in a validated risk score for predicting death in CCC (39). The reentrant circuit that causes VT and cardiac dysautonomia, a typical finding in CD, may be related to the pathogenesis and increased mortality risk associated with VT at rest in CD (40).

Conversely, the prognostic value of exercise-induced ventricular arrhythmias, especially in patients with CD, is less clear (41–44). Similar to our results, the presence of VT during the ET was associated with an increased risk of sudden death in a previous study including 69 CCC patients followed over a mean period of 24 months (45), being sudden death more frequently observed among patients with VT during the ET (*n* = 44) in comparison to those without VT (16 vs. 0%; *p* < 0.05).

During recovery from ET, our study demonstrated that ventricular arrhythmias (both VT and PVCs) were associated with increased mortality, which has already been previously demonstrated in non-CD individuals (46). Frolkis et al. investigated the association between ventricular arrhythmias during exercise and recovery with all-cause mortality in 29,244 patients with known or suspected coronary artery disease that underwent an ET. During a mean follow-up of 5.3 years, there were 1,862 deaths. After adjustment for potential confounders, ventricular arrhythmia during recovery was associated with a 60% increase on all-cause mortality risk, with no significant association with ventricular arrhythmias during exercise (47). Therefore, in this large cohort study, ventricular arrhythmia during recovery was a better predictor of an increased risk of death than ventricular arrhythmia during exercise.

The present study has some limitations. The retrospective design precluded us from obtaining information of some potential covariates that may result in potential residual confounding. In addition, censored information due to losses to follow-up (~20%) may have introduced some bias. However, the results obtained in the present study are in accordance with others that previously investigated the prognostic value of ET in other populations, reinforcing the importance of exercise-induced arrhythmias on prediction of mortality risk. Moreover, information about factors that may have changed over the time were not obtained (e.g., availability and access of healthcare, behavioral and sociodemographic risk factors, alternative treatments and point of intervention). However, we believe that these changes would have occurred in a similar frequency in the entire cohort, providing a non-differential error. Despite the relatively large sample size, the small number of some arrhythmias precluded us from performing a more detailed analysis about the influence of arrhythmias subtypes during the ET on mortality rates in CD patients (e.g., comparing sustained vs. non-sustained VT). In addition, the small percentage of patients with HF may indicate some degree of selection bias, limiting the extrapolation of the results to HF patients. Finally, the indirect evaluation of VO_2_max using both treadmill and cycle ergometer likely increases measurement error and may have contributed to the lack of association between functional capacity and mortality, even though most patients had performed the ET on a treadmill. On the other hand, our study is strengthened for long-term follow-up and the relatively high sample size, allowing a more reliable evaluation of the prognostic value of ET in patients with CD.

To conclude, our findings suggest that ET has important prognostic value for assessment of mortality risk in patients with CD. Increased DBP during exercise, presence of VT at rest, during exercise, and recovery, and PVCs during recovery were the variables independently associated with an increased mortality risk in patients with CD. The identification of individuals at higher mortality risk can facilitate the development of intervention strategies (e.g., close follow-up) that may potentially have an impact on longevity of patients with CD. Considering the potential prognostic value of ET in patients with CD, studies are necessary to assess if the inclusion of regular ET assessments would be beneficial for monitoring those with indeterminate or CCC to monitor for clinical evidence of worsening disease.

## Data availability statement

The raw data supporting the conclusions of this article will be made available by the authors upon reasonable request.

## Ethics statement

The studies involving human participants were reviewed and approved by Evandro Chagas National Institute of Infectious Disease Ethical Committee. The Ethics Committee waived the requirement of written informed consent for participation.

## Author contributions

RSS and MM contributed to the study design and drafted the manuscript. FM, IX, AS, RMS, AH-M, RSS, and MM contributed to the data acquisition. RSS, MM, and JF contributed to the data analysis and interpretation. All authors revised and approved the final version and agree with all aspects of the work in ensuring that questions related to the accuracy or integrity of any part of the work are appropriately investigated and resolved.

## Funding

The Fundação de Amparo à Pesquisa do Estado do Rio de Janeiro supported RSS during his master course for the development of this research project. This study was partially supported by the Coordenação de Aperfeiçoamento de Pessoal de Nível Superior– CAPES - Finance Code 001.

## Conflict of interest

The authors declare that the research was conducted in the absence of any commercial or financial relationships that could be construed as a potential conflict of interest.

## Publisher's note

All claims expressed in this article are solely those of the authors and do not necessarily represent those of their affiliated organizations, or those of the publisher, the editors and the reviewers. Any product that may be evaluated in this article, or claim that may be made by its manufacturer, is not guaranteed or endorsed by the publisher.

## Author disclaimer

The content of this manuscript is solely the responsibility of the authors and does not necessarily reflect the official views of the Veterans Health Administration, National Heart, Lung, and Blood Institute, National Institutes of Health or the United States Department of Health and Human Services.

## References

[1] World Health Organization. Chagas disease in Latin America: an epidemiological update based on 2010 estimates. Wkly Epidemiol Rec. (2015) 6:33–43. 25671846

[2] Pan American Health Organization. Guidelines for the Diagnosis and Treatment of Chagas Disease. Washington, D.C: PAHO (2019). 36 p.

[3] Lee BY Bacon KM Bottazzi ME Hotez PJ. Global economic burden of Chagas disease: a computational simulation model. Lancet Infect Dis. (2013) 13:342–8. 10.1016/S1473-3099(13)70002-1 23395248 PMC3763184

[4] Pérez-Molina JA Molina I. Chagas disease. Lancet. (2018) 391:82–94. 10.1016/S0140-6736(17)31612-4 28673423

[5] Dias JCP Ramos AN Jr Gontijo ED Luquetti A Shikanai-Yasuda MA Coura JR. 2 nd Brazilian consensus on Chagas Disease, 2015. Rev Soc Bras Med Trop. (2016) 49:3–60. 10.1590/0037-8682-0505-2016 27982292

[6] Costa HS Lima MMO Costa FSM da Chaves AT Nunes MCP Figueiredo PHS. Reduced functional capacity in patients with Chagas disease: a systematic review with meta-analysis. Rev Soc Bras Med Trop. (2018) 51:421–6. 10.1590/0037-8682-0158-2018 30133623

[7] Myers J Manish P Victor F Dat D Sara P Edwin AJ. Exercise capacity and mortality among men referred for exercise testing. N Engl J Med. (2002) 346:793–801. 10.1056/NEJMoa011858 11893790

[8] Fletcher GF Ades PA Kligfield P Arena R Balady GJ Bittner VA. Exercise standards for testing and training: a scientific statement from the American Heart Association. Circulation. (2013) 128:873–934. 10.1161/CIR.0b013e31829b5b44 23877260

[9] Sociedade Brasileira de Cardiologia. III Diretrizes da Sociedade Brasileira de Cardiologia Sobre Teste Ergométrico. Arq Bras Cardiol. (2015) 95:1–26. 10.1590/S0066-782X2010002400001 21340292

[10] Costa HS Lima MMO Figueiredo PHS Lima VP Ávila MR Menezes KKP de. Exercise tests in Chagas cardiomyopathy: an overview of functional evaluation, prognostic significance, and current challenges. Rev Soc Bras Med Trop. (2020) 53:e20200100. 10.1590/0037-8682-0100-2020 32638887 PMC7341832

[11] Nunes M do CP Rocha MOC Ribeiro ALP Colosimo EA Rezende RA Carmo GAA. Right ventricular dysfunction is an independent predictor of survival in patients with dilated chronic Chagas' cardiomyopathy. Int J Cardiol. (2008) 127:372–79. 10.1016/j.ijcard.2007.06.012 17689706

[12] Nunes M do CP Barbosa MM Ribeiro ALP Fenelon LMA Rocha MOC. Predictors of mortality in patients with dilated cardiomyopathy: relevance of Chagas disease as an etiological factor. Rev Esp Cardiol Engl Ed. (2010) 63:788–97. 10.1016/S1885-5857(10)70163-8 20609312

[13] Blackburn H Keys A Simonson E Rautaharju P Punsar S. The electrocardiogram in population studies: a classification system. Circulation. (1960) 21:1160–75. 10.1161/01.CIR.21.6.1160 13849070

[14] Prineas RJ Crow RS Zhang Z-M. The Minnesota Code Manual of Electrocardiographic Findings. London: Springer London (2010). 327 p.

[15] Lauer MS Francis GS Okin PM Pashkow FJ Snader CE Marwick TH. Impaired chronotropic response to exercise stress testing as a predictor of mortality. JAMA. (1999) 281:524–9. 10.1001/jama.281.6.524 10022108

[16] Cole CR Lauer MS. Heart-rate recovery immediately after exercise as a predictor of mortality. Heart. (1999) 341:1351–7. 10.1056/NEJM199910283411804 10536127

[17] Beltz NM Gibson AL Janot JM Kravitz L Mermier CM Dalleck LC. Graded exercise testing protocols for the determination of VO _2_ max: historical perspectives, progress, and future considerations. J Sports Med. (2016) 2016:1–12. 10.1155/2016/3968393 28116349 PMC5221270

[18] Bruce RA Kusumi F Hosmer D. Maximal oxygen intake and nomographic assessment of functional aerobic impairment in cardiovascular disease. Am Heart J. (1973) 85:546–62. 10.1016/0002-8703(73)90502-4 4632004

[19] Astrand P-O. Experimental Studies of Physical Working Capacity in Relation to Sex and Age. Copenhagen: Ejnar Munksgaard (1952).

[20] Jones NL Makrides L Hitchcock C Chypchar T McCartney N. Normal standards for an incremental progressive cycle ergometer test. Am Rev Respir Dis. (1985) 131:1–9. 3923878 10.1164/arrd.1985.131.5.700

[21] Naughton J Patterson J Fox SM. Exercise tests in patients with chronic disease. J Chronic Dis. (1971) 24:519–22. 10.1016/0021-9681(71)90040-3 5158764

[22] American Heart Association. Exercise Testing and Training of Apparently Healthy Individuals: A Handbook for Physicians. New York, NY: American Heart Association (1972). 40 p.

[23] Mora S Redberg RF Cui Y Whiteman MK Flaws JA Sharrett AR. Ability of exercise testing to predict cardiovascular and all-cause death in asymptomatic women. JAMA. (2003) 290:1600–7. 10.1001/jama.290.12.1600 14506119

[24] Keteyian SJ Patel M Kraus WE Brawner CA McConnell TR Piña IL. Variables measured during cardiopulmonary exercise testing as predictors of mortality in chronic systolic heart failure. J Am Coll Cardiol. (2016) 67:780–9. 10.1016/j.jacc.2015.11.050 26892413 PMC4761107

[25] Costa HS Lima MMO Figueiredo PHS Martinelli PM Camargos ER Chaves AT. Prognostic value of serum brain-derived neurotrophic factor levels in patients with Chagas cardiomyopathy. Mem Inst Oswaldo Cruz. (2018) 113:1–6. 10.1590/0074-02760180224 30133549 PMC6107101

[26] Costa HS Lima MMO Figueiredo PHS Chaves AT Nunes MCP da Costa Rocha MO. The prognostic value of health-related quality of life in patients with Chagas heart disease. Qual Life Res. (2019) 28:67–72. 10.1007/s11136-018-1980-7 30167935

[27] Mady C Cardoso RH Barretto AC da Luz PL Bellotti G Pileggi F. Survival and predictors of survival in patients with congestive heart failure due to Chagas' cardiomyopathy. Circulation. (1994) 90:3098–102. 10.1161/01.CIR.90.6.3098 7994859

[28] Ritt LE Carvalho AC Feitosa GS Pinho-Filho JA Andrade MVS Feitosa-Filho GS. Cardiopulmonary exercise and 6-min walk tests as predictors of quality of life and long-term mortality among patients with heart failure due to Chagas disease. Int J Cardiol. (2013) 168:4584–5. 10.1016/j.ijcard.2013.06.064 23871619

[29] Souza FC de C e Lorenzo AD Serra SM Colafranceschi AS. Chagas' cardiomyopathy prognosis assessment through cardiopulmonary exercise testing. Int J Cardiovasc Sci. (2015) 28:440–50. 10.5935/2359-4802.20150064

[30] Hasslocher-Moreno AM Saraiva RM Brasil PE Sangenis LH Xavier SS Sousa AS. Temporal changes in the clinical-epidemiological profile of patients with Chagas disease at a referral center in Brazil. Rev Soc Bras Med Trop. (2021) 54:1–7. 10.1590/0037-8682-0040-2021 34105626 PMC8186889

[31] Ventura-Garcia L Roura M Pell C Posada E Gascón J Aldasoro E. Socio-cultural aspects of chagas disease: a systematic review of qualitative research. PLoS Negl Trop Dis. (2013) 7:e2410. 10.1371/journal.pntd.0002410 24069473 PMC3772024

[32] Cucunubá ZM Okuwoga O Basáñez M-G Nouvellet P. Increased mortality attributed to Chagas disease: a systematic review and meta-analysis. Parasit Vectors. (2016) 9:1–42. 10.1186/s13071-016-1315-x 26813568 PMC4728795

[33] Maguire JH Hoff R Sherlock I Guimarães AC Sleigh AC Ramos NB. Cardiac morbidity and mortality due to Chagas' disease: prospective electrocardiographic study of a Brazilian community. Circulation. (1987) 75:1140–45. 10.1161/01.CIR.75.6.1140 3552307

[34] Sheps DS Ernst JC Briese FW Myerburg RJ. Exercise-induced increase in diastolic pressure: indicator of severe coronary artery disease. Am J Cardiol. (1979) 43:708–12. 10.1016/0002-9149(79)90067-5 425905

[35] Sydó N Sydó T Gonzalez Carta KA Hussain N Merkely B Murphy JG. Significance of an increase in diastolic blood pressure during a stress test in terms of comorbidities and long-term total and CV mortality. Am J Hypertens. (2018) 31:976–80. 10.1093/ajh/hpy080 29767671

[36] Webb AJS. Progression of arterial stiffness is associated with midlife diastolic blood pressure and transition to late-life hypertensive phenotypes. J Am Heart Assoc Cardiovasc Cerebrovasc Dis. (2020) 9:1–11. 10.1161/JAHA.119.014547 31902329 PMC6988142

[37] Nürnberger J Dammer S Opazo Saez A Philipp T Schäfers RF. Diastolic blood pressure is an important determinant of augmentation index and pulse wave velocity in young, healthy males. J Hum Hypertens. (2003) 17:153–8. 10.1038/sj.jhh.1001526 12624604

[38] Thanassoulis G Lyass A Benjamin EJ Larson MG Vita JA Levy D. Relations of exercise blood pressure response to cardiovascular risk factors and vascular function in the Framingham heart study. Circulation. (2012) 125:2836–43. 10.1161/CIRCULATIONAHA.111.063933 22572915 PMC3636551

[39] Rassi A Rassi A Little WC Xavier SS Rassi SG Rassi AG. Development and validation of a risk score for predicting death in Chagas' heart disease. N Engl J Med. (2006) 355:799–808. 10.1056/NEJMoa053241 16928995

[40] Barbosa MPT Carmo AAL do Rocha MO da C Ribeiro ALP. Ventricular arrhythmias in Chagas disease. Rev Soc Bras Med Trop. (2015) 48:4–10. 10.1590/0037-8682-0003-2014 25714933

[41] Morise AP. Exercise testing in nonatherosclerotic heart disease: hypertrophic cardiomyopathy, valvular heart disease, and arrhythmias. Circulation. (2011) 123:216–25. 10.1161/CIRCULATIONAHA.109.914762 21242508

[42] Marine JE Shetty V Chow GV Wright JG Gerstenblith G Najjar SS. Prevalence and prognostic significance of exercise-induced nonsustained ventricular tachycardia in asymptomatic volunteers. J Am Coll Cardiol. (2013) 62:595–600. 10.1016/j.jacc.2013.05.026 23747767 PMC3800197

[43] Zicker F Zicker EMS Oliveira JJ Netto JCA Oliveira RM Smith PG. Exercise electrocardiogram tests in manual workers with and without antibodies to *Trypanosoma cruzi*: a population-based study. Trans R Soc Trop Med Hyg. (1990) 84:787–91. 10.1016/0035-9203(90)90081-O 2128980

[44] Pedrosa RC Salles JHG Magnanini MMF Bezerra DC Bloch KV. Prognostic value of exercise-induced ventricular arrhythmia in Chagas' heart disease: Chagas' heart disease: exercise-induced arrhythmia. Pacing Clin Electrophysiol. (2011) 34:1492–97. 10.1111/j.1540-8159.2011.03171.x 21797898

[45] de Paola AA Gomes JA Terzian AB Miyamoto MH Martinez Fo EE. Ventricular tachycardia during exercise testing as a predictor of sudden death in patients with chronic chagasic cardiomyopathy and ventricular arrhythmias. Heart. (1995) 74:293–5. 10.1136/hrt.74.3.293 7547025 PMC484021

[46] Dewey FE. Ventricular arrhythmias during clinical treadmill testing and prognosis. Arch Intern Med. (2008) 168:225–34. 10.1001/archinte.168.2.225 18227372

[47] Frolkis JP. Frequent ventricular ectopy after exercise as a predictor of death. N Engl J Med. (2003) 348:781–90. 10.1056/NEJMoa022353 12606732

