# Supplementary material for: The association of exercise test variables with long-term mortality in patients with chronic Chagas disease
Source: Front Med (Lausanne). 2022 Sep 20;9:972514. doi: 10.3389/fmed.2022.972514 (PMC9530636; doi:10.3389/fmed.2022.972514)
Supplement: Supplementary file 1 [file Table_1.pdf]

Supplemental Table 1. Characteristics of patients according to loss to follow-up status (n=232).

Variable	Median (IQR25% to 75%) or Frequency (%)		*p-value*
	Completers (n=178; 76.7%)	Losses to follow-up (n= 54; 23.3%)	
Follow-up time (years)	25.2 (15.7 to 28.3)	5.1 (3.0 to 10.3)	**<0.001**
Age (years)	47.0 (41.0 to 52.0)	40.0 (32.0 to 48.0)	**<0.001**
Women	86 (48.3)	30 (55.6)	0.351
Race			
White	98 (55.1)	28 (51.8)	0.679
Non-white	80 (44.9)	26 (48.1)	
Region of origin according to prevalence			
Non-endemic Chagas disease area	9 (5.1)	2 (3.7)	0.677
Low Chagas disease prevalence area	7 (4.0)	5 (9.3)	0.124
Medium Chagas disease prevalence area	54 (30.5)	18 (33.3)	0.695
High Chagas disease prevalence area	107 (60.5)	29 (53.7)	0.378
Body mass index (Kg/m^2)	24.7 (22.4 to 27.6) n=118	25.1 (22.4 to 26.6) n=30	0.943
Hypertension	49 (27.5)	8 (14.8)	0.057
Diabetes Mellitus	4 (2.2)	0 (0.0)	0.266
Dyslipidemia	28 (15.7)	9 (16.7)	0.869
Non-CD cardiomyopathy	6 (3.4)	3 (5.6)	0.466
Heart failure	10 (5.6)	3 (5.6)	0.986
Stroke	1 (0.6)	0 (0.0)	0.581
Clinical presentation of CD			
Indeterminate	82 (46.1)	31 (57.4)	0.144
Cardiac	96 (53.9)	23 (42.6)	0.144
Digestive	4 (2.2)	1 (1.8)	0.861
VO_2max ($mL.kg^{-1}.min^{-1}$)	31.3 (24.6 to 38.4)	34.8 (31.3 to 41.4)	**0.015**
LVEF (Teicholz, %)	64.0 (58.0 to 69.0)	63.0 (58.0 to 69.0)	0.787
ST-segment abnormalities	7 (3.9)	1 (1.8)	0.463
Supraventricular arrhythmias			
Resting PACs	11 (6.2)	4 (7.4)	0.748
Resting SVT[ł]	0 (0.0)	0 (0.0)	1.00

Exercise PACs	29 (16.3)	6 (11.1)	0.351
Exercise SVT	1 (0.6)	0 (0.0)	0.581
Recovery PACs	23 (12.9)	6 (11.1)	0.725
Recovery SVT	1 (0.6)	1 (1.8)	0.369
Ventricular arrhythmias			
Resting PVCs	59 (33.1)	10 (18.5)	**0.039**
Resting VT	4 (2.2)	1 (1.8)	0.861
Exercise PVCs	91 (51.1)	26 (48.1)	0.702
Exercise VT	17 (9.5)	5 (9.3)	0.949
Recovery PVCs	69 (38.7)	16 (29.6)	0.222
Recovery VT	10 (5.6)	5 (9.3)	0.341

CD: Chagas disease; LVEF: Left Ventricular Ejection Fraction; PACs: Premature atrial contractions; SVT: Supraventricular tachycardia; PVCs: Premature ventricular complexes; VT: Ventricular tachycardia

Supplemental Table 2. Sensitivity analysis for the association between ET variables and death, including the type of ergometer (treadmill or cycle ergometer) as a covariate.

	**Adjusted***		
**Variable**	**HR**	**95%CI**	***p-value***
VO_2max (ml/kg/min)	0.98	(0.95 to 1.01)	0.136
FAI (%)	1.01	(0.99 to 1.02)	0.109
Resting HR (bpm)	1.00	(0.99 to 1.01)	0.938
Maximal HR (bpm)	1.00	(0.99 to 1.01)	0.794
ΔHR during exercise (bpm)	1.00	(0.99 to 1.01)	0.837
Chronotropic deficit (HRmax<85%)	0.94	(0.61 to 1.45)	0.789
Recovery HR at 1st minute (bpm)	1.00	(0.99 to 1.01)	0.438
ΔHR during Recovery at 1st minute (bpm)	1.00	(0.99 to 1.02)	0.431
ΔHR Recovery ≤ 12 bpm	1.25	(0.65 to 2.40)	0.505
Resting SBP (mmHg)	1.00	(0.99 to 1.02)	0.362
Resting DBP (mmHg)	1.00	(0.98 to 1.02)	0.920
Maximal SBP (mmHg)	1.00	(0.99 to 1.01)	0.657
Maximal DBP (mmHg)	**1.02**	**(1.00 to 1.03)**	**0.038**
ΔSBP during Exercise (mmHg)	0.99	(0.98 to 1.00)	0.374
ΔDBP during Exercise(mmHg)	**1.03**	**(1.01 to 1.05)**	**0.005**
Double Product (mmHg.bpm)	1.00	(0.99 to 1.00)	0.962
ST-segment abnormalities	0.87	(0.27 to 2.81)	0.823
Supraventricular arrhythmias			
Resting PACs	1.42	(0.63 to 3.19)	0.401
Resting SVT[ł]	-	-	-
Exercise PACs	0.98	(0.55 to 1.74)	0.935
Exercise SVT	1.68	(0.21 to 13.26)	0.620
Recovery PACs	1.21	(0.69 to 2.11)	0.503
Recovery SVT	1.34	(0.17 to 10.51)	0.778

Ventricular arrhythmias			
Resting PVCs	1.30	(0.84 to 2.02)	0.230
Resting VT	**3.94**	**(1.13 to 13.76)**	**0.031**
Exercise PVCs	1.05	(0.69 to 1.60)	0.805
Exercise VT	**2.76**	**(1.44 to 5.28)**	**0.002**
Recovery PVCs	**2.07**	**(1.33 to 3.24)**	**0.001**
Recovery VT	**2.60**	**(1.14 to 5.93)**	**0.023**

VO_2: Oxygen Consumption; FAI: Functional Aerobic Impairment; SBP: Systolic Blood Pressure; DBP: Diastolic Blood Pressure; HR: Heart Rate; MET: Metabolic Equivalent; AHA: American Heart Association; ΔHR: heart rate variation; ΔSBP: Systolic Blood Pressure Variation; ΔDBP: Diastolic Blood Pressure Variation; LVEF: Left Ventricular Ejection Fraction; PACs: Premature atrial contractions; SVT: Supraventricular tachycardia; PVCs: Premature ventricular complexes; VT: Ventricular tachycardia

* Cox regression model adjusted for age, sex, race, classification of CD (indeterminate, CCC without heart failure, and CCC with heart failure), LVEF, and presence of comorbidities (arterial hypertension, diabetes mellitus, dyslipidemia, non-CD cardiomyopathy, or stroke), and type of ergometer

[ƚ] Non-valid HR estimates due to zero counts in at least one of the groups

Estimates **in bold** were statistically significant
